# The Neurotrophic Receptor Ntrk2 Directs Lymphoid Tissue Neovascularization during *Leishmania donovani* Infection

**DOI:** 10.1371/journal.ppat.1004681

**Published:** 2015-02-24

**Authors:** Jane E. Dalton, Amy C. Glover, Laura Hoodless, Eng-Kiat Lim, Lynette Beattie, Alun Kirby, Paul M. Kaye

**Affiliations:** 1 Centre for Immunology and Infection, Department of Biology and Hull York Medical School, University of York, York, United Kingdom; 2 Jack Birch Unit, Department of Biology, University of York, York, United Kingdom; University of Massachusetts Medical School, UNITED STATES

## Abstract

The neurotrophic tyrosine kinase receptor type 2 (Ntrk2, also known as TrkB) and its ligands brain derived neurotrophic factor (Bdnf), neurotrophin-4 (NT-4/5), and neurotrophin-3 (NT-3) are known primarily for their multiple effects on neuronal differentiation and survival. Here, we provide evidence that Ntrk2 plays a role in the pathologic remodeling of the spleen that accompanies chronic infection. We show that in *Leishmania donovani*-infected mice, Ntrk2 is aberrantly expressed on splenic endothelial cells and that new maturing blood vessels within the white pulp are intimately associated with F4/80^hi^CD11b^lo^CD11c^+^ macrophages that express Bdnf and NT-4/5 and have pro-angiogenic potential in vitro. Furthermore, administration of the small molecule Ntrk2 antagonist ANA-12 to infected mice significantly inhibited white pulp neovascularization but had no effect on red pulp vascular remodeling. We believe this to be the first evidence of the Ntrk2/neurotrophin pathway driving pathogen-induced vascular remodeling in lymphoid tissue. These studies highlight the therapeutic potential of modulating this pathway to inhibit pathological angiogenesis.

## Author Summary

Visceral leishmaniasis (VL), a globally important parasitic disease responsible for over 40,000 deaths p.a., results in pronounced changes in splenic organisation associated with impaired immune function and persistent parasite infection. We have previously shown that receptor tyrosine kinase (RTKi) inhibitors can restore splenic architecture and improve immunocompetence, and that mononuclear phagocytes (MPs) are involved in this process. Here, we provide evidence that neurotrophin receptor Ntrk2 (also known as TrkB) plays a role in the pathologic remodeling of the spleen that accompanies experimental *Leishmania donovani*-infection. We show that following infection of mice with *L.donovani*, Ntrk2 is expressed on splenic endothelial cells that are closely associated with F4/80^hi^CD11b^lo^CD11c^+^ macrophages expressing Ntrk2 ligands. Administration of the Ntrk2 antagonist ANA-12 to infected mice significantly inhibited compartment-specific vascular remodeling of the spleen. This study expands our understanding of the pathogenesis of experimental VL and also demonstrates the potential of Ntrk2/Bdnf as targets for treatment of infection-induced vascular remodeling.

## Introduction

Disruption in lymphoid tissue organisation is evident in a wide array of chronic inflammatory disorders, including cancer, infectious disease, psoriasis, liver disorders, autoimmune and metabolic diseases, and the persistence of vascular remodelling has been shown to impair immune responses and promote the chronicity of inflammation [1–4]. It is perhaps no surprise, therefore, that anti-angiogenic drugs are becoming an important therapeutic option for an array of chronic diseases and disorders, including those of infectious origin. HIV, malaria, schistosomiasis and leishmaniasis all promote chronic inflammation and remodelling of lymphoid organs [5–8], and murine models of these diseases have been used extensively to study the mechanistic basis of stromal cell and vascular remodelling and their consequences in terms of immune dysfunction and disease progression [7,9–11].

Splenomegaly is a defining characteristic of the parasitic disease visceral leishmaniasis (VL) and like many examples of splenomegaly, VL is associated with a remodeling of splenic architecture [4,12]. In murine experimental VL, enlargement of red pulp vessels and neovascularization of the white pulp are prominent. Previously, we demonstrated that administration of the broad spectrum receptor tyrosine kinase inhibitor (RTKi) sunitinib maleate to *Leishmania donovani*-infected mice halted progressive vascular remodeling, reduced mononuclear phagocyte (MP) number and improved the efficacy of immune-dependent drugs [4,13,14]. Compartment-specific remodeling of the red pulp vasculature was subsequently identified as a function of Ly6C^+^ inflammatory monocytes [14], whereas these cells played no apparent role in regulating white pulp neovascularisation or the loss of follicular dendritic cells (FDC) and fibroblastic reticular cells (FRC), suggesting alternate and independent mechanisms exist to regulate these processes.

Neurotrophins and their receptors are novel targets for angiogenic therapies [15,16]. For example, Bdnf is recognised as an angiogenic factor, being critical for the establishment of cardiac vasculature during development [17,18], and for promoting Ntrk2-dependent skeletal muscle neoangiogenesis in adult ischemic hind limbs [19]. However, it is the limited tissue expression of the neutrophin receptors, such as Ntrk2, which makes them so desirable as targets for therapy, with the potential for aberrant expression in pathological conditions in other tissues being largely overlooked.

MPs are recognized as playing a significant role in inflammation-induced angiogenesis [2,20] and MP are known to be a source of Bdnf and NT-4/5 with their production being increased upon inflammatory stimulation [21,22]. Chronic parasitic and bacterial infections have also been associated with elevated levels of Bdnf/BDNF [23–25]. For example, serum levels of BDNF were significantly higher in patients suffering from chronic Chagas disease compared to healthy controls [25]. However, the cellular source(s) of neurotrophin were not determined in these studies. A causal link, therefore, between neurotrophin expression and infection-induced, macrophage-directed angiogenesis has not been previously reported.

Here, we report that Ntrk2 is aberrantly expressed on endothelial cells that form newly emerging vessels within the white pulp during *L. donovani* infection and that expression of Bdnf is up regulated in a sub-population of MPs with phenotypic similarity to resident tissue macrophages. Together, these observations have important implications for understanding the pathogenesis of VL and illustrate the potential for aberrant expression of angiogenic receptors and their ligands during inflammation.

## Results

### Characterisation of MPs in *Leishmania* infected mice

**Fig 1:**
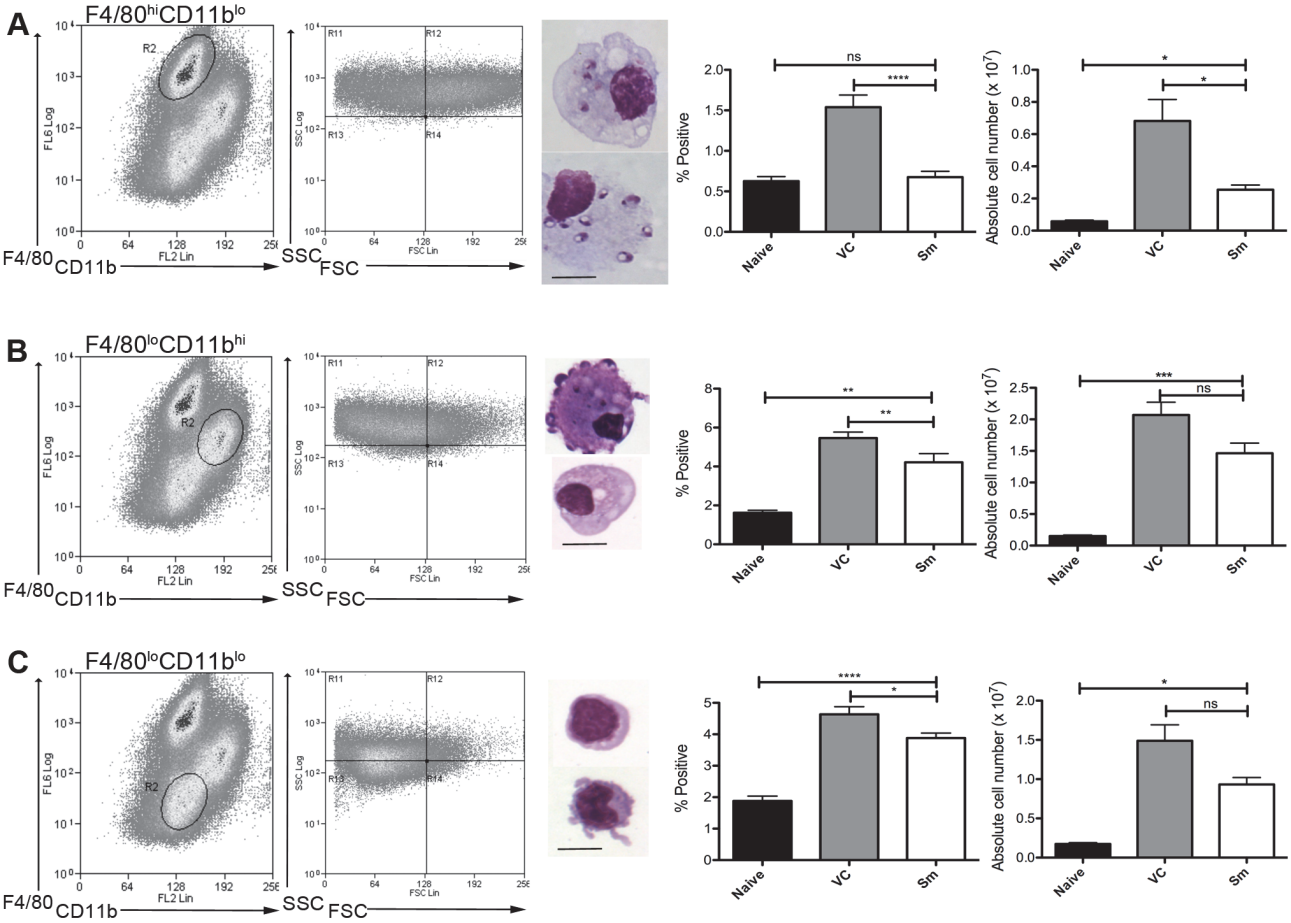
Sensitivity of mononuclear phagocytes in *L.donovani* infected mice to RTKi treatment. CD11c^+^MHCII^+^ MPs in d28 *L. donovani* infected mice were distinguished on the basis of F4/80 and CD11b expression, forward/side scatter profile and morphology into: (A) large CD11c^+^MHCII^+^F4/80^hi^CD11b^lo^ (F4/80^hi^CD11b^lo^) cells with macrophage-like morphology, of which ∼80% harbored intracellular parasites; (B) slightly smaller CD11c^+^MHCII^+^F4/80^lo^CD11b^hi^ (F4/80^lo^CD11b^hi^) cells with classic macrophage morphology, of which <5% harbored parasites; (C) much smaller CD11c^+^MHCII^+^F4/80^lo^CD11b^lo^ (F4/80^lo^CD11b^lo^) cells with dendritic cell-like morphology and no observable parasites. Representative dot plots show pre-sorted populations with ellipsoid sort gates based on F4/80 and CD11b expression. Scale bar in micrographs = 10microns. The frequency and absolute numbers of each population is given in the right hand panels in naïve mice, infected mice and infected mice treated orally with sunitinib (Sm) for 7 days. P values = * <0.05, ** <0.008, ***<0.001, ns = not significant.

To determine whether MPs were involved in the process of white pulp remodeling, we first characterized MPs in the spleen of sunitinib-treated and control mice after the onset of splenomegaly (d28 post infection;[4]). Three distinct populations of CD11c^+^MHCII^+^ MPs were identified in infected mice based on CD11b, F4/80 and forward/side scatter profile and morphology: F4/80^hi^CD11b^lo^ cells (large, macrophage-like morphology; ∼80% containing parasites; Fig. 1A); F4/80^lo^CD11b^hi^ cells (smaller, classic macrophage morphology, <5% infected; Fig. 1B); and F4/80^lo^CD11b^lo^ cells (small, dendritic cell morphology, no parasites; Fig. 1C). These MP populations increased in number 9–14 fold in infected compared to naïve mice. Although administration of sunitinib reduced the number of all populations, this was most marked for the F4/80^hi^CD11b^lo^ cells (Fig. 1A-C, right panels).

**Fig 2:**
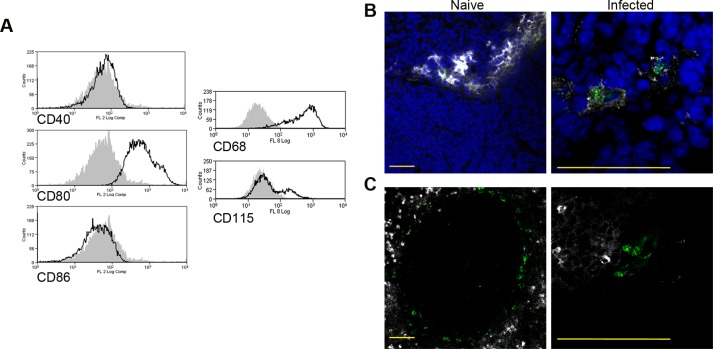
Phenotypic analysis of F4/80^hi^CD11b^lo^cells. Splenocytes isolated from *L.donovani* infected mice at 28 days post infection were stained with a panel of myeloid cell markers. CD11c^+^MHCII^+^F4/80^hi^CD11b^lo^ MPs were positive for CD80, CD68 and a small proportion (15%) expressed CD115 (A: isotype control, filled grey histogram). Strong SIGNR1 (white) and FITC-dextran (green) labeling co-localise in the marginal zone of naïve mice (B), whereas in infected mice FITC-dextran^+^ (green) cells had low expression of SIGNR1 (white). FITC-dextran^+^ (green) cells were negative for GR1 (white) in both naïve and infected mice (C). Scale bars = 100 microns.

As the sensitivity of F4/80^hi^CD11b^lo^ MPs to sunitinib treatment correlated with inhibition of white pulp neovascularisation, we further characterized these cells in both untreated infected and sunitinib-treated infected mice. Phenotypically, F4/80^hi^CD11b^lo^ MPs from both groups of mice were CD68^+^Ly6G/C^-^ CD80^+^ SIGNR1^lo^CD115^+/-^ (Fig. 2A-C), suggesting that these MPs might be resident rather than inflammatory monocytes / macrophages. To further characterize these cells, we used an in-house MP-targeted oligoarray (consisting of >500 genes representing multiple GO pathways; S1 Table) to identify genes differentially expressed (DE) in F4/80^hi^CD11b^lo^ MPs vs. a reference population of F4/80^hi^CD11b^lo^ peritoneal MPs. The top DE gene was *Slc40a1*, an iron export protein expressed at high levels by tissue resident red pulp macrophages (Table 1), with many other DE genes being associated with pro-inflammatory and IFNγ-regulated responses, e.g. *Cxcl9, Il18, Fpr2* and *Ccl4* (Table 2 and S2 Table).

**Table 1:** Altered gene expression in F4/80^hi^CD11b^lo^ cells compared to control macrophages.

Gene	Fold change	Gene	Fold change
*Slc40a1*	6.44	*Ticam1*	-11.36
*Cxcl9*	3.77	*Tgfb2*	-3.28
*Il18*	3.42	*Spi1*	-3.13
*Tgfb3*	3.13	*Il1r1*	-2.72
*Fpr2*	2.85	*Il1rn*	-2.60
*Ccl4*	2.74	*Xcl1*	-2.57
*Arhgdia*	2.72	*Ccr1*	-2.38
*Il1b*	2.72	*Sp1*	-2.31
*Mr1*	2.71	*Arg1*	-2.29
*Clec4n*	2.69	*Hif1an*	-2.23

**Table 2:** Comparison of growth factor mRNA abundance in F4/80^hi^CD11b^lo^ cells compared to other MP populations isolated from *L. donovani*-infected spleens.

Gene	Fold change relative to:		
	**F4/80** ^**lo**^ **CD11b** ^**hi**^	**CD11b** ^**lo**^ **F4/80** ^**lo**^	**CD11c** ^**neg**^
*Bmp4*	44.32	6.68	17.88
*Bdnf*	21.26	2.36	2.38
*Cxcl12*	18.01	6.20	10.70
*Bmp2*	15.35	8.22	5.82
*Bmp6*	13.10	2.81	4.50
*Tgfa*	12.47	5.47	4.82
*Fgf11*	9.85	18.51	15.24
*Kitl*	8.22	15.67	4.47
*Tdgf1*	6.92	3.51	3.41
*Ntf5*	6.32	4.06	4.06
*Fgf1*	6.19	4.32	4.20
*Igf1*	5.82	15.67	5.62
*Pdgfa*	5.35	5.35	4.89
*Il18*	3.43	16.56	4.38
*Tgfb3*	-3.92	-15.89	-10.56
*Artn*	-2.75	-3.43	-10.34
*Pgf*	-2.10	-4.29	-5.78
*Csf1*	-2.04	-2.91	-4.14

### F4/80^hi^ CD11b^lo^ MPs are associated with blood endothelial cells in the splenic white pulp

**Fig 3:**
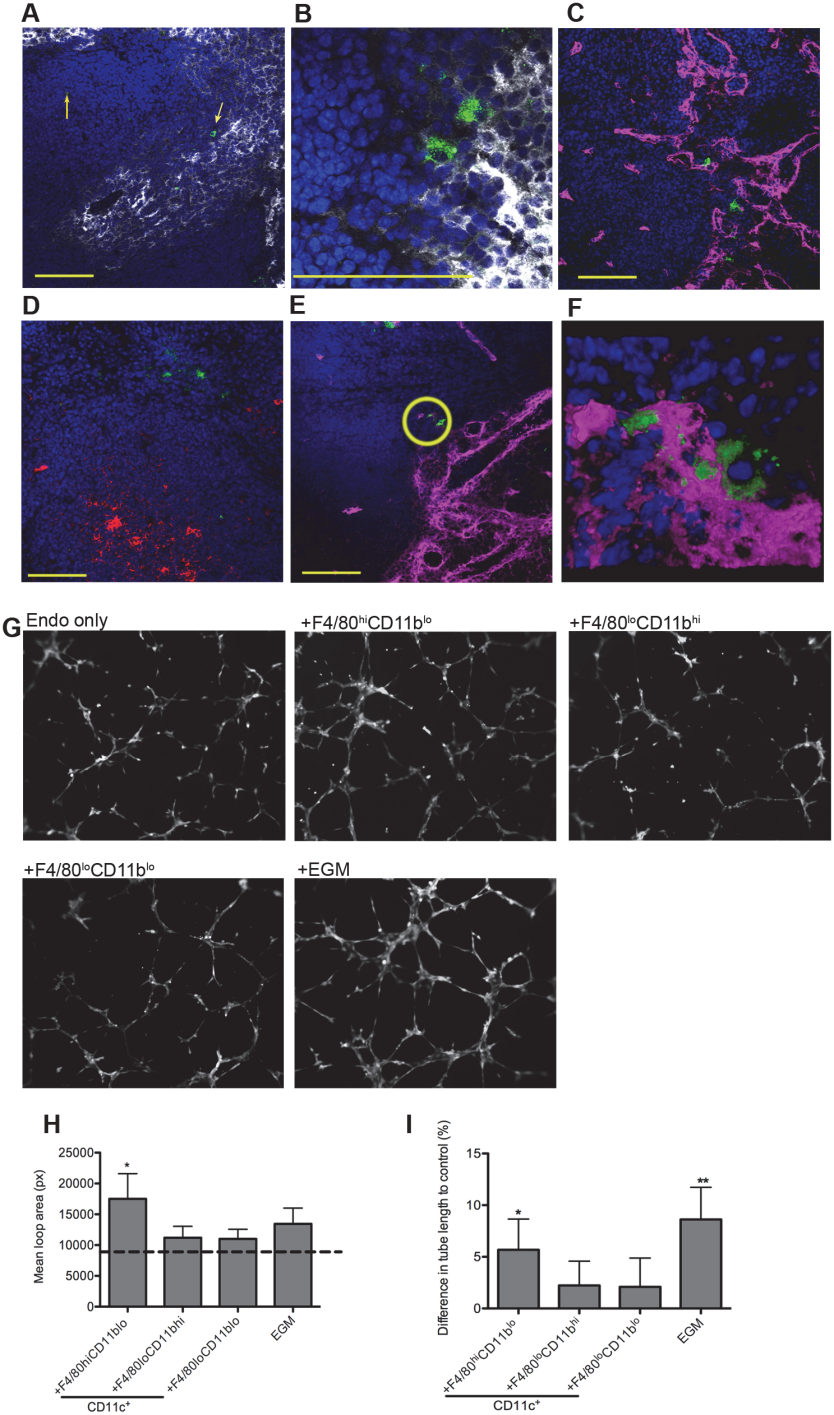
F4/80^hi^CD11b^lo^ MPs are located in close proximity to white pulp vasculature and possess angiogenic properties. F4/80^hi^CD11b^lo^ cells (FITC-dextran, green; yellow arrows) identified in fresh frozen sections as located either in or bordering the white pulp (A,B). Red pulp F4/80^+^ macrophages are also shown (white). F4/80^hi^CD11b^lo^ cells (FITC-dextran, green) were found in close association with endothelial cells (C, E; Meca-32, magenta) but not follicular dendritic cells (D; FDCM1, red). High magnification image of area depicted by yellow circle in e (F). All sections were counterstained with DAPI (blue). Scale bars = 100 microns. F4/80^hi^CD11b^lo^ cells, but not other splenic MPs tested, drive SVEC4–10 endothelial cell tube formation on a gelled basement membrane extract (G). Representative images are shown. An optimised cocktail of growth factors (EGM) was used as a positive control. Quantitative analysis of SVEC4–10 mean loop area (H) and difference in tube length (I), in the presence of each MP population or control growth factors. Mean loop area in the absence of growth factors or MPs is shown as a dotted line in h. *p = 0.05, **p = 0.02. Images were analysed using WimTube software and data are expressed as mean ± SEM from at least three independent experiments.

F4/80^hi^ macrophages were described 30 years ago immediately adjacent to arterioles in the periarteriolar lymphoid sheath [26,27]. We localized F4/80^hi^CD11b^lo^ MPs in situ, taking advantage of their expression of SIGNR1, a receptor for the uptake of FITC-dextran [28], and the absence of SIGNR1^hi^ marginal zone macrophages in *L. donovani* infected mice [11]. 74% of cells labeled intra-vitally with FITC-dextran were CD11c^+^F4/80^hi^CD11b^lo^ (gating strategy in S1 Fig.). In situ, F4/80^hi^CD11b^lo^ FITC-dextran^+^ MPs were located in either the white pulp region of the spleen or adjacent to the MZ (Fig. 3A, B). F4/80^hi^CD11b^lo^ MPs were most commonly (87.5%±7.2) found in association with Meca32^+^ vessels adjacent to the MZ or no more than a distance of two cell nuclei away (8.3% ± 8), and located predominantly at vessel junctions (Fig. 3C, D). x-y-z- reconstructions confirmed that they were closely associated with smooth muscle actin (SMA)-positive cells (S1 Video). Finally, 3D-rendering of z-plane images confirmed the presence of F4/80^hi^CD11b^lo^ MPs tightly associated with vessel junctions and vasculature that protruded into the white pulp from the marginal sinus (Fig. 3E-F and S2 Video).

### F4/80^hi^ CD11b^lo^ MPs are pro-angiogenic

The close association of F4/80^hi^CD11b^lo^ MPs with vessels penetrating the white pulp suggested that there maybe a causal relationship with vascular remodeling. To determine the angiogenic potential of F4/80^hi^CD11b^lo^ MPs, we used SVEC4–10 mouse endothelial cells in an *in vitro* tube formation assay. SVEC4–10 cells cultured in the presence of an optimized cocktail of growth factors (EGM) migrated, directionally align and formed tube networks to a greater extent than cells cultured in in basal media (EBM) (Fig. 3G). Addition of F4/80^lo^CD11b^hi^ and F4/80^lo^CD11b^lo^ cells had negligible tube promoting activity (<20% of maximal activity; difference in tube length of 2.2±2.3% and 2.1±2.7%, respectively vs. EBM control), whereas F4/80^hi^CD11b^lo^ MPs significantly enhanced mean loop area and tube length (5.7±3.0% and 8.6±3.1% for F4/80^hi^ CD11b^lo^ MPs and EGM respectively, compared to EBM control; Fig. 3G-I). Hence, of the splenic MPs tested, only F4/80^hi^CD11b^lo^ MPs displayed angiogenic activity *in vitro*.

**Fig 4:**
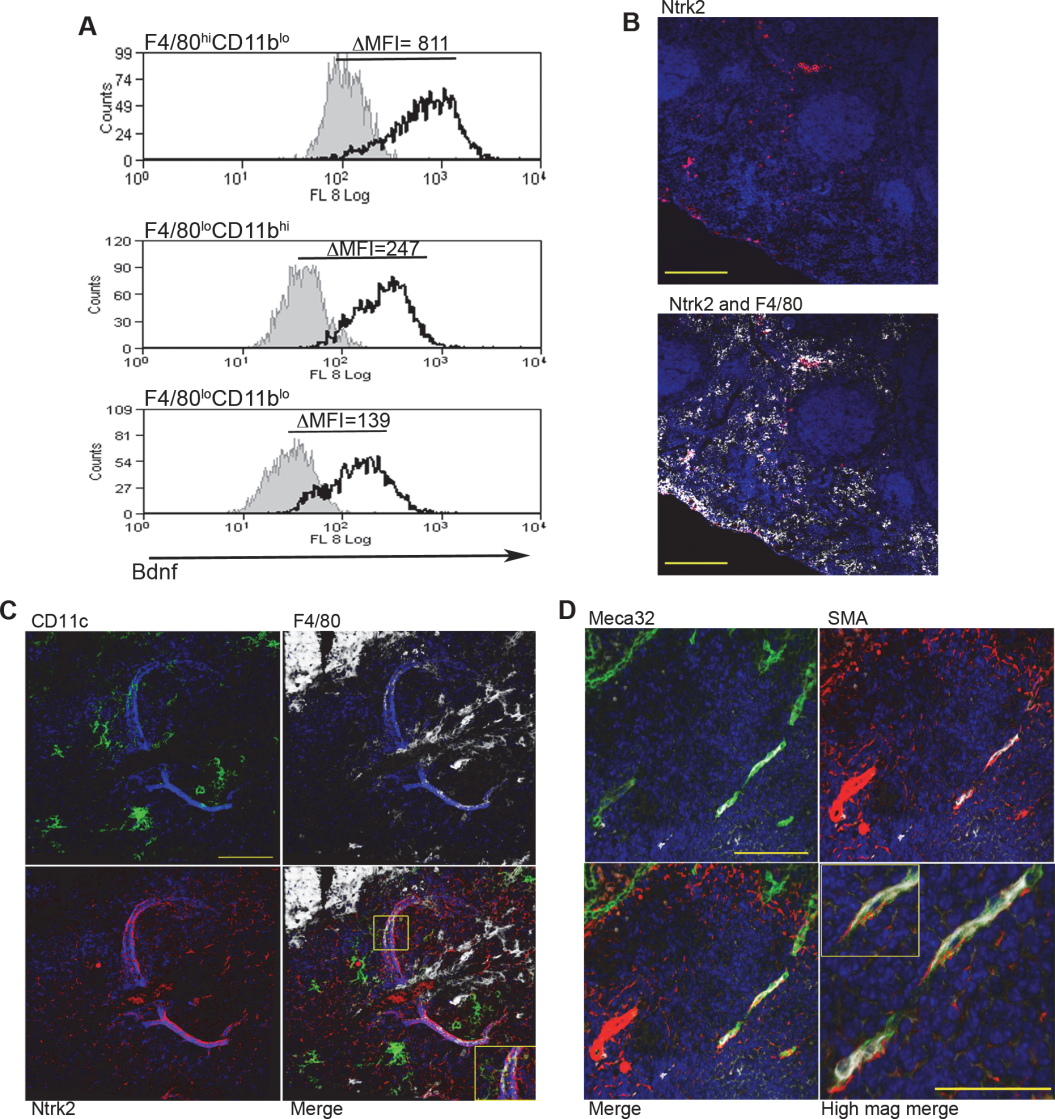
Infection-induced expression of Bdnf and Ntrk2 in spleen. Bdnf (black line) was expressed at significantly higher levels by F4/80^hi^CD11b^lo^ cells compared to the other MP populations tested, as revealed by delta median fluorescence intensity levels (ΔMFI) using intracellular flow cytometry (A). Isotype control staining is highlighted by the solid grey histogram. Ntrk2 (red) expression in naïve mouse spleen is found in a subset of splenic red pulp macrophages (F4/80^+^, white) in naïve mouse spleen (B). Scale bar = 200microns. In *L. donovani* infected mice splenic white pulp vessels expressed Ntrk2 (C: red). F4/80^+^ (white) CD11c^+^ (green) MPs are found in close association with Nrtk2^+^ vessels (C: merge and inset). Endothelial cells (Meca-32, green) but not smooth muscle actin-positive cells (SMA, red) in white pulp express Nrtk2 (white, D and inset). All sections were stained with DAPI (blue). Scale bars = 100 microns and 50 microns in high magnification merge image.

To identify factors associated with the angiogenic activity of these MPs, we studied the expression of 84 angiogenesis-related genes using PCR array. 69% (58/84) of genes were DE (2-fold cut-off) in F4/80^hi^CD11b^lo^ MPs relative to F4/80^lo^CD11b^hi^ MPs (46 up, 12 down), 50% (42/84) were DE relative to F4/80^lo^CD11b^lo^ MPs (28 up, 14 down) and 55% (46/84) were DE relative to non-adherent CD11c^-^ splenocytes (28 up, 18 down). 21% (18/84; 14 up, 4 down) were DE relative to all groups (Table 2). These included several members of the *Tgf* family and known ligands for sunitinib, including *Pdgf, Kitl Artn, Pgf* and *Csf1*. Neurotrophic factors *Fgf11, Bdnf* and *Ntf5* (neurotrophin 4/5) were all up regulated in F4/80^hi^CD11b^lo^ MPs (Table 1). F4/80^hi^CD11b^lo^ MPs also expressed higher levels of Bdnf protein compared to other MP populations studied (ΔMFI = 811 vs ΔMFI = 247 and ΔMFI = 139; Fig. 4A). Although we cannot rule out a causal link between intracellular infection of F4/80^hi^CD11b^lo^ MPs by *L. donovani* amastigotes and their Bdnf expression, *Bdnf* gene expression was not enhanced following *L. donovani* infection of a variety of other MPs *in vitro* [29] and *in vivo* [30]. In summary, these data demonstrate that F4/80^hi^CD11b^lo^ MPs in infected mice: i) are appropriately positioned in the tissue; ii) express neurotrophins with known angiogenic activity; and iii) are pro-angiogenic *in vitro*.

### Nrtk2 expression on vasculature following infection in vivo

Spleen tissue sections were analyzed for the expression of Ntrk2, the receptor for Bdnf to investigate a possible role for this pathway in vascular remodeling *in vivo*. In the naïve spleen, Ntrk2 expression was restricted to a population of F4/80^+^ red pulp MPs, corresponding to previous reports [31] (Fig. 4B). Expression of Ntrk2 was not altered at d1 post infection, suggesting that induction of Ntrk2 was not the result of inflammatory responses that immediately follow infection. Induced expression was seen in the marginal zone area at day 7 post infection and staining within the white pulp area was beginning to become apparent at day 14 (S2 Fig.). In spleens from chronically infected mice, Nrtk2 was aberrantly expressed on stromal cells, with white pulp vessels showing the highest intensity of staining for Ntrk2 (Fig. 4C). Ntrk2^+^ vessels were found to be in extremely close association with CD11c^+^F4/80^+^ MPs (Fig. 4C). Ntrk2 expression has been reported on endothelial cells and smooth muscle cells in developing and adult cardiac tissue [18,32]. Co-staining with Meca32 indicated that Ntrk2 was expressed on splenic endothelial cells, but vessels associated with high levels of SMA staining did not express detectable levels of Ntrk2 (Fig. 4D). As SMA acquisition is a marker of vessel maturity [33], these data suggest that Ntrk2 is up regulated only on developing or immature blood vessels.

### Pharmacologic blockade of Ntrk2 inhibits neovascularisation

**Fig 5:**
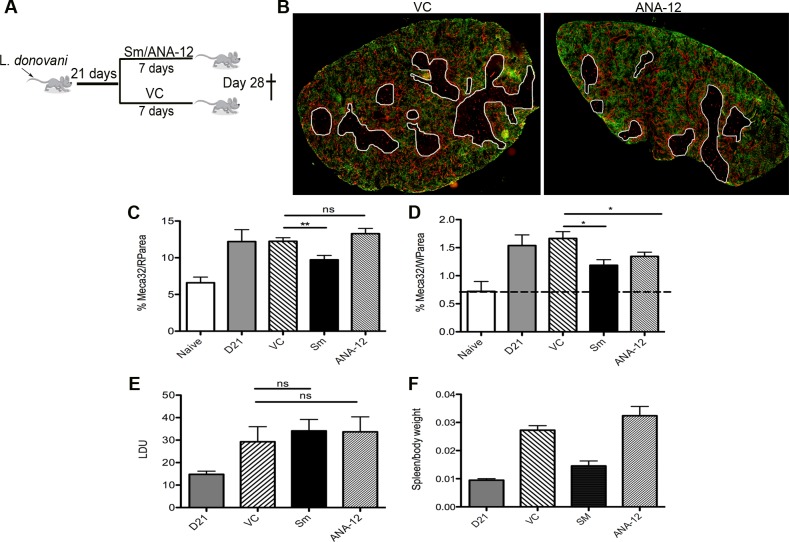
White pulp neovascularization during chronic infection is dependent on Nrtk2. Drug treatment schedule: 21 days post infection with *L. donovani*, mice were treated with ANA-12, sunitinib maleate (Sm) or vehicle control (VC), daily for 7 days (A). Spleen sections from control and drug-treated mice were stained for endothelial cells (Meca-32, red) and F4/80 (green) to identify boundary of the red and white pulp (white lines). Representative whole slide scanned images are shown (B). The proportion of red pulp area (C) occupied by endothelial cells (Meca-32) and expression of Meca32 relative to total white pulp area (D) was determined by computer-assisted morphometry. Data are expressed as mean ± SEM of at least two independent experiments where n = 5 for each treatment. ** p<0.02 *p<0.05. Spleens were examined after drug treatment for parasite burdens (E) and size/body weight ratio (F).

**Fig 6:**
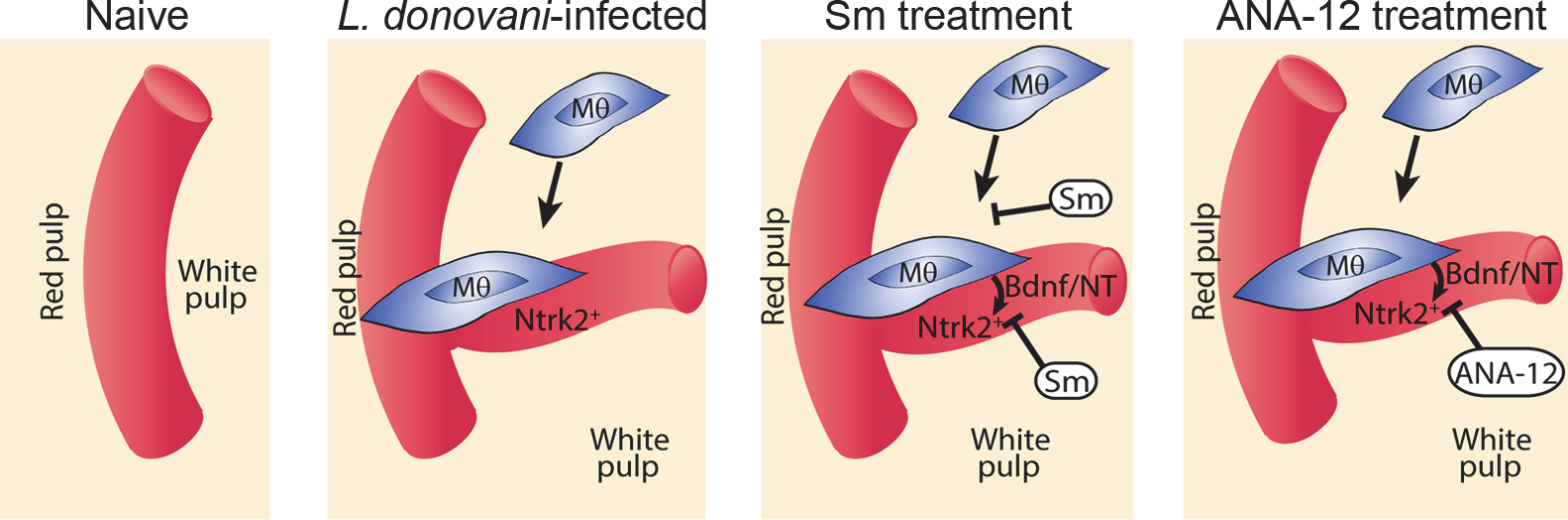
Schematic diagram of the effects of treatment with RTK inhibitors during *L.donovani* infection. Cartoon to depict processes inhibited by the broad spectrum RTKi (Sm) and the selective Nrtk2 inhibitor (ANA-12) during infection-associated neovascularization of white pulp.

Finally, to determine whether this pathway played a functional role in vascular remodeling, we inhibited Ntrk2 signaling *in vivo* using the small molecule antagonist ANA-12 [34]. Mice were treated for 7 days i.p. with sunitinib or ANA-12 commencing on day 21 p.i. [4] (Fig. 5A). Quantitative image analysis indicated that sunitinib [4] but not ANA-12 inhibited red pulp vascular remodeling (Fig. 5B and C). In contrast, both sunitinib and ANA-12 significantly inhibited white pulp neovascularization (50%±10 and 34%±8 reduction vs. vehicle control, respectively; Fig. 4B and D). ANA-12 did not alter splenic parasite burden, as was also the case for sunitinib (Fig. 5E). Unlike sunitinib, however, ANA-12 treatment had no significant effect on spleen size (Fig. 5F) suggesting that splenomegaly and red pulp vascular remodeling are linked. Similarly, MP frequency was unaltered following ANA-12 treatment (1.15±0.1% vs 1.0±0.1% for F4/80^hi^CD11b^lo^ MPs, 5.83±0.5% vs 5.82±0.3% for F4/80^lo^CD11b^hi^ MPs and 5.25±0.5% vs 5.3±1.5% for F4/80^lo^CD11b^lo^ MPs, comparing ANA-12 to vehicle). Collectively, these data demonstrate that targeting Ntrk2 signaling *in vivo* selectively inhibits ongoing white pulp neovascularization, without affecting MP infiltration and/or proliferation or splenomegaly and red pulp vascular remodeling (Fig. 6).

## Discussion

Chronic inflammation results from sustained immune mediated inflammatory responses leading to significant tissue destruction and / or remodeling, but the cells and mechanisms involved in these processes, especially in the context of infectious disease, are not fully understood. Our data provides the first evidence that the neurotrophic receptor Ntrk2 and its ligands play a role in mediating pathological vascular remodeling. First, we show that Ntrk2 is aberrantly expressed on lymphoid tissue (white pulp) vasculature in the spleen of mice with chronic infection-associated inflammation. This contrasts with the largely CNS-restricted expression of Ntrk2 expression observed in healthy adult tissues. Second, we have identified a population of splenic F4/80^hi^ CD11b^lo^ MPs that express Ntrk2 ligands and possess all the characteristics needed to drive white pulp vascular remodeling. Third, we demonstrate that a selective antagonist of Ntrk2 inhibits ongoing white pulp neovascularization.

Angiogenesis and inflammation have long been thought of as co-dependent processes and there is increasing evidence that disruption to vasculature prolongs and intensifies the inflammatory response [35]. It has been shown that MPs are recruited to sites of neoangiogenesis and support neovascularization by releasing angiogenic factors, including VEGF, PDGF, FGF and metalloproteinases [36–38]. Recently, evidence has suggested that neuronal factors such as neurotrophic factors, ephrins, bone morphogenetic proteins (BMPs) and their receptors [19,39,40], can also play critical roles as angiogenic regulators. A study by Kermani et al highlighted that Bdnf promoted neovascularization in ischemic adult limbs and this correlated with the expression of Ntrk2 on endothelial cells. In agreement with that study, we found enrichment of Bdnf in F4/80^hi^ CD11b^lo^ MPs and could directly show that these cells were closely associated with endothelial cells expressing Ntrk2. The expression of Ntrk2 by splenic endothelial cells was only observed in chronic infection and was not evident in the steady-state.

ANA-12 is a low molecular weight heterocyclic compound that was identified using a novel structure-based in silico screening technique as a selective Ntrk2 inhibitor [34]. In mice, ANA-12 has been shown to have anti-depressant and anti-anxiolytic activity [34] and it alters cocaine-mediated behaviour in rats [41]. Ntrk2 is also targeted, less selectively by other RTKi’s in the clinic or in early stage clinical development e.g. Lestaurtinib (for neuroblastoma;[42]) and PLX7486 (pancreatic adenocarcinoma; ClinicalTrials.gov identifier: NCT01804530). If aberrant expression of Ntrk2 is confirmed in other settings of chronic inflammation associated with pathogenic angiogenesis, Ntrk2-selective drugs may provide a new therapeutic option for treatment of these conditions. Importantly, the potential for aberrant expression and function of this neurotrophic receptor, as shown here, also highlights the need to consider inflammation when designing organ-specific therapeutics.

Developmentally, tissue macrophages are known to have functions in matrix remodeling [43], epithelial proliferation and outgrowth [44], angiogenesis and tissue organization [45,46]. It has been suggested that in chronic disease, the developmental activities of macrophages are dysregulated and that these activities contribute to disease pathology [47]. This is evident in some models of cancer where depletion of macrophages or inhibition of macrophage access to the tumor site inhibits tumor growth and angiogenesis[48–51]. Gene expression signatures of tumour-associated macrophages revealed enrichment in genes associated with developmental functions including matrix remodeling, and angiogenesis [52]. Consistent with these findings, F4/80^hi^ CD11b^lo^ MPs isolated from the spleen of *L. donovani*-infected mice revealed enrichment in expression of genes functionally related to cancer and cell survival. These cells displayed angiogenic properties in vitro and given their spatial relationship with emerging blood vessels, it is highly likely that these cells are playing a role in neovascularisation in vivo.

Splenic macrophages are themselves a heterogeneous population, of which the immunological phenotype and immunological function of all populations has still not been extensively explored, particularly during chronic inflammation. The CD11c^+^MHCII^+^F4/80^hi^ CD11b^lo^ MP population identified here that is expanded during *L. donovani* infection is rare in the spleen of uninfected mice, making it hard to draw direct comparisons with any function of these cells in the steady-state. F4/80^+^ macrophages found in the white pulp of the spleen were originally described 30 years ago in adult mice to be immediately adjacent to arterioles in the periarteriolar lymphoid sheath [26], and later studies defined these MPs as having high expression of the F4/80 antigen [27]. Although the low expression of SIGNR1 suggests that these MPs may have some relationship to marginal zone macrophages, cell-tracking studies of MZM in the spleens of infected mice suggest that most bone fide MZM are lost from the spleen during infection [11]. A formal identification of the relationship of the CD11c^+^MHCII^+^F4/80^hi^ CD11b^lo^ MP population described here to different splenic MP populations present in the steady state would require lineage tracking studies that are beyond the scope of the current manuscript.

Finally, the impact of ANA-12 on tissue remodeling was highly selective compared to the impact we have previously observed using a broader spectrum RTKi, sunitinib maleate. Following sunitinib administration, the ongoing remodeling of red and white pulp vasculature is halted and follicular dendritic cell and fibroblastic reticular cell networks are largely restored [4]. In contrast, the impact of ANA-12 administration was selective for white pulp neovascularization. Likewise, whereas red pulp vasculature is remodeled largely by Ly6C^+^ inflammatory monocytes, white pulp neovascularization appears to reflect the more local behavior of MPs with a more resident-like phenotype. Further studies will be required to determine whether selective blockade of white pulp neovascularization using ANA-12 has any selective effects on immune function than might support its use in combination therapy for disease such as leishmaniasis, where lymphoid tissue remodeling is so prominent. However, in common with broader spectrum RTKi, placental expression of Ntrk2 and the influence of maternal energetic status on placental Ntrk2 expression[53] would likely be an impediment to development of this and similar drugs for the treatment of women of childbearing age.

In conclusion, we have shown that selected features of pathogenic angiogenesis induced by chronic infection are mediated through the interaction of a neurotrophic receptor with ligands produced by local MPs. Our data provide new insights into the role of the neurotrophins and their receptors in inflammation, and further characterize the exquisite compartment specific nature of tissue remodeling processes. The role of macrophage expression of Bdnf in other chronic infectious and inflammatory disease settings clearly warrants further study.

## Materials and Methods

### Ethics statement

All animal care and experimental procedures were regulated under the Animals (Scientific Procedures) Act 1986 (revised under European Directive 2010/63/EU) and were performed under UK Home Office License (Ref # PPL 60/4377) and with approval from the Animal Procedures and Ethics Committee of the Department of Biology, University of York.

### Experimental infection and treatment

Female C57BL6 CD45.1 and CD45.2 mice were obtained from Charles River UK, housed under specific pathogen-free conditions and used at 6–10 weeks of age. The Ethiopian strain of *L. donovani* (LV9) was maintained by passage in RAG-2^-/-^ mice. Mice were infected by injecting 3x10^7^ amastigotes i.v. via the lateral tail vein (total n = 20 for each treatment experiment). Animals were then allocated to treatment groups. Sunitinib maleate (35mg/kg; Sequoia Research Products Ltd) (n = 5) or ANA-12 (1mg/kg; Sigma) (n = 5) was administered daily via oral gavage or intraperitoneally for 7d. Vehicle-treated mice received citrate-buffered saline, pH 3.5 (Sm) or PBS/1% DMSO (ANA-12) (n = 5). A sample size of 5 for each experiment was required to detect a 30% change in vascularization at 80% power based on previous data [4]. For FITC-dextran uptake, 70,000 MW FITC-dextran was injected i.v. at 10mg/ml one hour before animals were sacrificed.

### Cell culture of SVEC4–10 cells

Murine endothelial cells (ATCC) were grown in DMEM media supplemented with 10% heat inactivated FCS in an atmosphere of 95% air and 5% CO_2_ at 37°C in plastic flasks. At confluence, the cells were subcultured at a 1:3 ratio and used at passage numbers three through to ten. Cells were tested for mycoplasma contamination using MycolAlert mycoplasma detection kit (Lonza, Cat No: LT07–418)

### Microarray and pathway analysis

Custom microarrays with 568 gene probes, including 32 control probes were printed in-house. The 536 test probes were chosen to provide insight into the various functions and signalling pathways of macrophages.

Total RNA was isolated from splenic macrophages from *L.donovani*-infected mice (experimental samples) and from naive pooled peritoneal macrophages (reference sample). Amplified reference and experimental RNA fluorescently labelled with Cy3 or Cy5 were combined in equal amounts on custom printed microarray slides and hybridised overnight at 42°C. The average experimental: reference ratios were compared by *t*-tests. Microarray data was analysed using Ingenuity Pathways Analysis. The web-based pathways analysis tool IPA (Ingenuity Systems, www.ingenuity.com) was used to identify biological and molecular networks. Knowledge coming from published, peer-reviewed scientific publications is stored in the Ingenuity Pathways Knowledge Base (IPKB) and is continuously updated, this web-based tool then allows for the mapping of gene expression data into relevant pathways based on their functional annotation and known molecular interactions. The genes considered to have been differentially regulated to a significant extent when comparing F4/80^hi^CD11b^lo^ cells with conventional peritoneal macrophages were uploaded into IPA along with the gene identifiers and corresponding fold change values. In the *network analysis*, networks of these genes are then algorithmically generated based on their connectivity. The *functional analysis of a network* identified the biological functions and/or diseases that were most significant to the genes in the network, and *the functional analysis of the entire data set* identified the biological functions and/or diseases that were most significant to the data set.

### Flow cytometry and cell sorting

Mononuclear cells were prepared from the spleens of C57BL6 *L. donovani* infected mice. Isolated cells were labelled with F4/80 (clone: BM8), CD11c (clone: N418), CD80 (clone: 16–10A1), CD86 (clone: GL1), CD40 (clone: IC10), CD115 (clone: AFS98), MHCII (clone: M5/114), CD11b (clone: M1/70), Gr-1 (Ly6C/G clone: RB6–8C5) all purchased from eBioscience and CD68 (clone FA-11) and CD169 (clone MOMA-1) were purchased from Serotec. Intracellular cytokine staining was performed following surface staining on fixed cell (2% paraformaldehyde) and then permeabilised with 0.5% saponin. Cells were analyzed using a cyAn flow cytometer and analyzed using Summit software (Beckman Coulter). Cells were sorted based on forward and side scatter and expression of PE or PE-Cy7 conjugated anti-CD11c, Alexa Fluor 450 anti-MHCII, Alexa Fluor 488 conjugated anti-CD11b and Alexa Fluor 647 anti-F480 on a MoFlo cell sorter (Beckman Coulter). Sorted cells were then used for tube formation assays, analysed for mRNA expression or morphological analysis was carried out with approximately 3000 sorted cells spun onto glass slides, fixed in methanol and stained with Giemsa. Light microscopy was conducted on a Zeiss Axioplan and imaged with an Optronics CCD camera using MagnaFire software (Optronics).

### Confocal microscopy

Phenotypic analysis of MPs was also carried out on splenic frozen sections. Sections (6–10μm) from FITC-dextran injected, infected or uninfected mice were acetone fixed and labeled with biotin conjugated Gr-1 or biotin conjugated F4/80 and Alexa fluor 647 SIGNR1 (eBioscience).

Thicker sections (10–30μm) were used to assess the location of MPs within the spleen. Sections were labeled with a purified rat anti mouse pan endothelial cell antigen antibody (Biolegend, clone Meca-32) and/or a Cy3 labelled monoclonal antibody against α-Smooth Muscle Actin (α-SMA) (Sigma, Clone 1A4) were used to identify endothelial cells or vessels. Rat anti-mouse FDC antibody (FDCMI) (BD Pharmingen) was used to detect follicular dendritic cells. Fluorochrome conjugated goat anti-rat antibody (Invitrogen) were used for detection of purified antibodies. Sections were counterstained with DAPI and mounted in Pro-long Gold anti-fade (Invitrogen) and visualized using a Carl Zeiss upright LSM META 510, inverted LSM META 710 confocal microscope or a Carl Zeiss Axio Scan.Z1 digital slide scanner with brightfield and 4-colour fluorescence.

### Tube formation assay

Angiogenesis assays were carried out as described previously [54]. Isolated splenocytes from chronically infected mice were first enriched for MPs by plastic adherence for 1hr at 37oC in RPMI (10% FCS) before cell sorting. Proliferating SVEC4–10 cells (1.5 x10^4^ cells) were added to sorted populations of MPs or non-adherent cells (2 x 10^4^) in endothelial basal media-2, with no FCS, (EBM-2; Lonza) and then plated onto growth factor-reduced Cultrex basement membrane (BME; Trevigen) coated 96 well plates. Negative controls of SVEC4–10 cells in EBM-2 and positive controls of SVEC4–10 cells in endothelial cell growth media-2 (EGM-2; Lonza) containing FCS and growth supplements (SingleQuot kit; Lonza) were included. Tube formation was assayed 4 hours after plating. Images were taken using a Zeiss inverted fluorescent microscope with x5 objective. All conditions were set up in each experiment in triplicate and imaged. Images were then uploaded onto http://www.wimasis.com/ for independent blind analysis using WimTube, web-based image analysis software, which quantitatively evaluated the generation of new vessels. Assays were analyzed by WimTube Quantitative Image Analysis.

### Growth factor PCR array

Total RNA was extracted from the sorted cells using the RNeasy RNA isolation kit (Qiagen), and then quantified using a Nanodrop ND-100. For the PCR array, 200ng of total RNA was reverse transcribed using RT^2^ First Stand Kit (Qiagen), and cDNA was directly added to PCR Master mix containing SYBR green. The mixtures were then aliquoted into 96-well PCR array plates, to profile the expression of 84 growth factor pathway-related genes using a mouse growth factor signalling pathway RT^2^ Profiler PCR array (PAMM-041Z, Qiagen) according to the manufacturer’s instructions. The array also included 6 housekeeping genes and 3 RNA as internal controls. Arrays were run on an ABI 7300HT qPCR instrument equipped with SDS 2.3 software, using RT^2^ SYBR Green/ROX qPCR master mix (Qiagen). Data analysis was done by the 2^-ΔΔCt^ method on the manufacturer’s Web portal http://www.SABiosciences.com/pcrarraydataanalysis.php

### Statistics

Data are expressed values as means ± SEM. Comparison was performed using the unpaired Student’s t-test (for data following a Gaussian distribution) and the Mann-Whitney test (for data that did not assume Gaussian distribution). D’Agostino and Pearson omnibus normality test was used to test for Gaussian distribution. A probability of less than 5% (P < 0.05) was considered to be statistically significant. All statistical analyses were performed with Prism v5.01 (GraphPad, Inc.) software.

## Supporting Information

S1 Video F4/80^hi^CD11b^lo^ cells are closely associated with splenic stroma. F4/80^hi^CD11b^lo^ cells in day 28 infected mice were intravitally-labeled with FITC-dextran (green). 30-micron spleen sections were stained with for smooth muscle actin (SMA) and Z stacks acquired in 1micron steps. 3D rendering was performed using VolocityTM software (Improvision). The movie was exported at 10 frames per second (fps) and shows the SMA^+^ stromal network (red) with tightly associated FITC-dextran^+^ cells. Nuclear staining was performed with DAPI (blue).
(MOV)

S2 Video F4/80^hi^CD11b^lo^ cells are tightly associated with junctions of vasculature emanating from the white pulp border. F4/80^hi^CD11b^lo^ cells in day 28 infected mice were intravitally-labeled with FITC-dextran (green). 30-micron spleen sections were stained with Meca32 (red) to highlight vasculature. FITC-dextran^+^ cells were found in very close association with vessels, in particular with the branch point of vessels entering the white pulp. Z stacks were acquired in 1micron steps and the 3D rendering done in VolocityTM software (Improvision). The movie was exported at 10 frames per second (fps). Nuclear staining is shown in blue using DAPI.
(MOV)

S1 Fig F4/80^hi^CD11b^lo^ cells preferentially take up low molecular weight FITC-dextran in *L.donovani* infected spleens. Mice at 28 days post infection with *L.donovani* were intravitally labeled with 70kDa FITC-dextran 10mins before sacrifice. Flow cytometric analysis of splenocytes positive for FITC staining confirmed that the majority of these cells were CD11c^+^ MHCII^+^ F4/80^hi^ and CD11b^lo^.
(TIF)

S2 Fig Kinetics of expression of Ntrk2 in the spleen of *L. donovani* infected mice. Mice were infected with *L. donovani* and at time indicated, spleen sections were stained for Ntrk2. A-D represent merged images (blue, DAPI; green, F4/80; red, Ntrk2, white, CD11c. E-H show Ntrk2 with DAPI counterstaining only.
(TIFF)

S1 Table Gene list for probes included on oligo-array. (XLSX)

S2 Table Top 300 probes by fold change in F4/80^hi^CD11b^lo^ cells compared to control macrophages. (XLSX)
