# Supplementary figures and images for: The Neurotrophic Receptor Ntrk2 Directs Lymphoid Tissue Neovascularization during Leishmania donovani Infection
Source: PLoS Pathog. 2015 Feb 24;11(2):e1004681. doi: 10.1371/journal.ppat.1004681 (PMC4339582; doi:10.1371/journal.ppat.1004681)

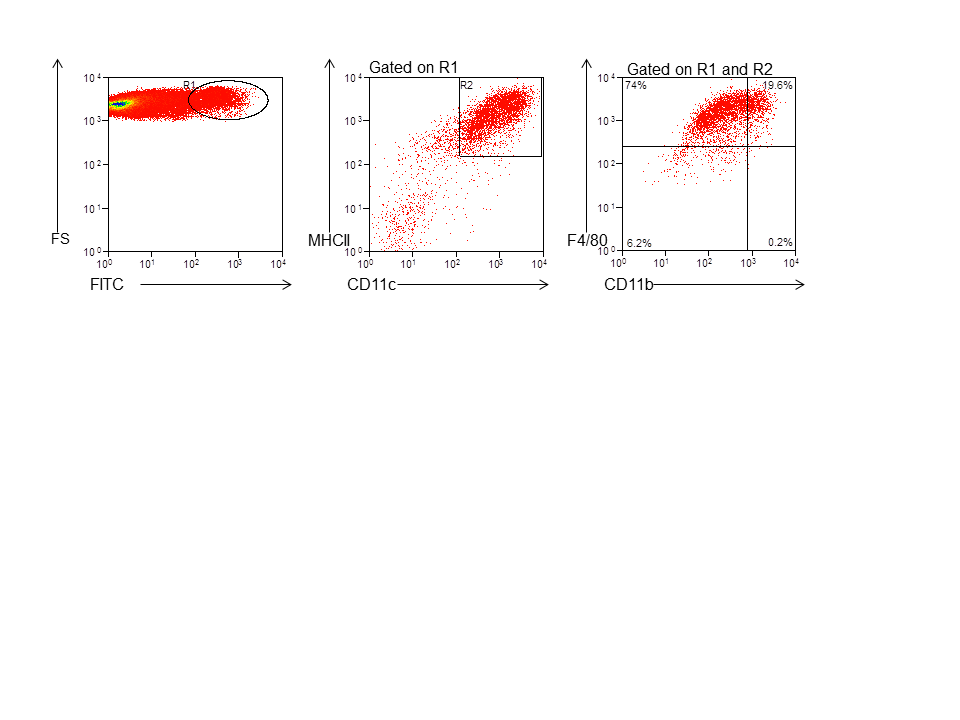

Supplement: S1 Fig — Mice at 28 days post infection with L.donovani were intravitally labeled with 70kDa FITC-dextran 10mins before sacrifice. Flow cytometric analysis of splenocytes positive for FITC staining confirmed that the majority of these cells were CD11c+ MHCII+ F4/80hi and CD11blo. (TIF) [file ppat.1004681.s003.tif]

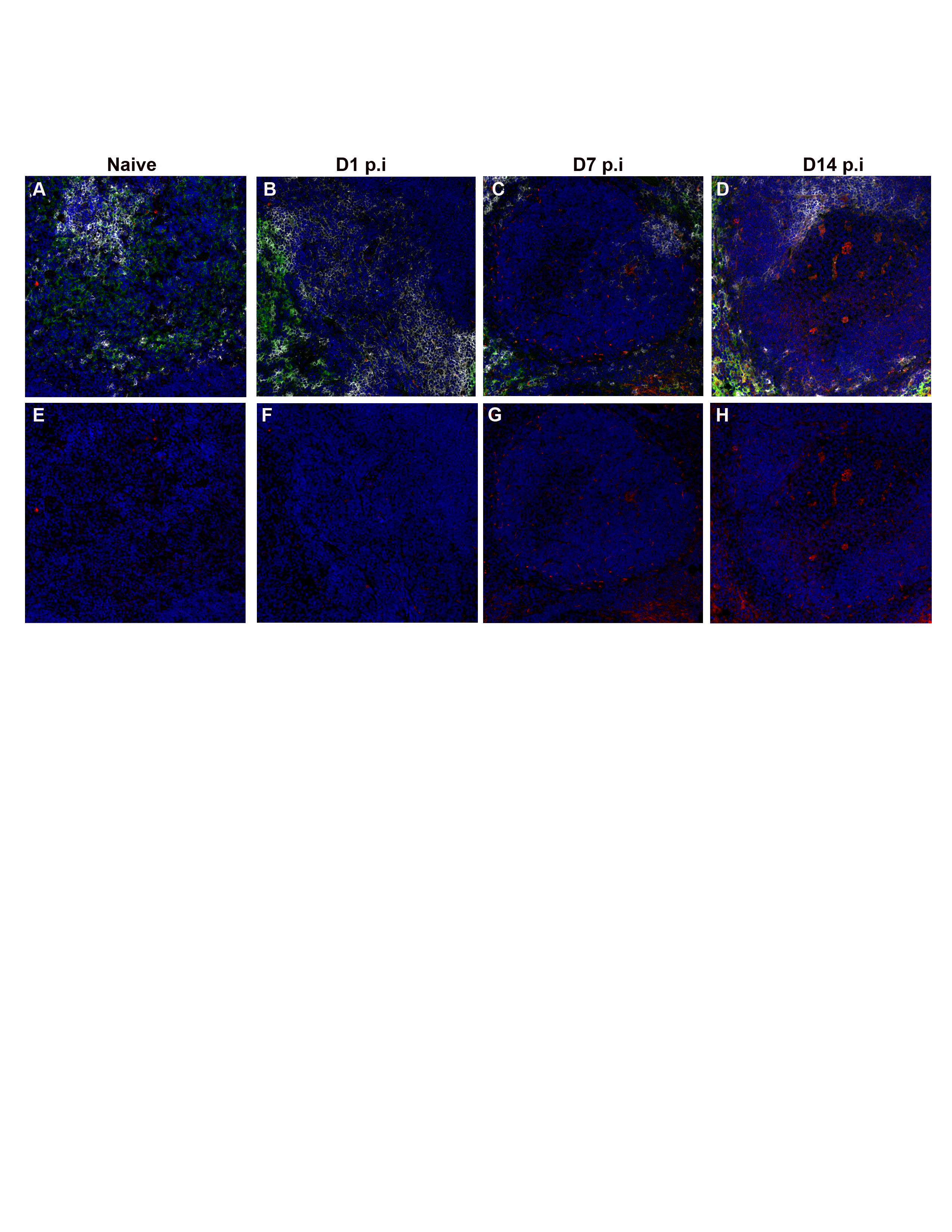

Supplement: S2 Fig — Mice were infected with L. donovani and at time indicated, spleen sections were stained for Ntrk2. A-D represent merged images (blue, DAPI; green, F4/80; red, Ntrk2, white, CD11c. E-H show Ntrk2 with DAPI counterstaining only. (TIFF) [file ppat.1004681.s004.tiff]
